# Antidiabetic Activity of *Artemisia amygdalina* Decne in Streptozotocin Induced Diabetic Rats

**DOI:** 10.1155/2014/185676

**Published:** 2014-05-21

**Authors:** Khalid Ghazanfar, Bashir A. Ganai, Seema Akbar, Khan Mubashir, Showkat Ahmad Dar, Mohammad Younis Dar, Mudasir A. Tantry

**Affiliations:** ^1^Department of Biochemistry, Faculty of Biological Sciences, University of Kashmir, Srinagar 190 006, India; ^2^Drug Standardisation Research Unit, Regional Research Institute of Unani Medicine (CCRUM), University of Kashmir, Naseem Bagh, Srinagar 190 006, India; ^3^Centre of Research for Development, University of Kashmir, Srinagar 190 006, India

## Abstract

*Artemisia* species have been extensively used for the management of diabetes in folklore medicine. The current study was designed to investigate the antidiabetic and antihyperlipidemic effects of *Artemisia amygdalina*. Petroleum ether, ethyl acetate, methanol, and hydroethanolic extracts of *Artemisia amygdalina* were tested for their antidiabetic potentials in diabetic rats. The effect of extracts was observed by checking the biochemical, physiological, and histopathological parameters in diabetic rats. The hydroethanolic and methanolic extracts each at doses of 250 and 500 mg/kg b. w significantly reduced glucose levels in diabetic rats. The other biochemical parameters like cholesterol, triglycerides, low density lipoproteins (LDL), serum creatinine, serum glutamate pyruvate transaminase (SGPT), serum glutamate oxaloacetate transaminase (SGOT), and alkaline phosphatise (ALP), were found to be reduced by the hydroethanolic and methanolic extracts. The extracts also showed reduction in the feed and water consumption of diabetic rats when compared with the diabetic control. The histopathological results of treated groups showed the regenerative/protective effect on **β**-cells of pancreas in diabetic rats. The current study revealed the antidiabetic potential of *Artemisia amygdalina* being effective in hyperglycemia and that it can effectively protect against other metabolic aberrations caused by diabetes in rats, which seems to validate its therapeutic traditional use.

## 1. Introduction

Diabetes mellitus is a metabolic disorder characterized by a loss of glucose homeostasis, with disturbances of carbohydrate, fat, and protein metabolism resulting from defects in insulin secretion, insulin action, or both [1]. Diabetes mellitus is represented by hyperglycemia, lipidaemia, and oxidative stress; it predisposes affected individuals to long-term complications affecting the eyes, skin, kidneys, nerves, and blood vessels [2]. Diabetesis prevalent in all parts of the world and rapidly increasing worldwide. The estimated number of adults living with diabetes has soared to more than 371 million, having a prevalence of 8.3% [3]. India has the more than 63 million of diabetic persons [3]. Despite considerable progress in the treatment of diabetes by oral hypoglycaemic agents, search for newer drugs continues because the existing synthetic drugs have several limitations and harmful effects [4, 5]. Therefore, managing diabetes without any side effects is still a challenging task for health care providers [6]. Hence, the studies are being conducted for finding more efficient, safer, and less expensive hypoglycaemic agents. Herbal medicines have ever been used and claimed as antidiabetic agents but very less are available on commercially formulated forms [7].

Ethnomedicine is a promising field of research in Kashmir, as the valley grows varied medicinal and aromatic plants including those used in curing various diseases [8, 9]. It has been reported that there are 220 medicinal plant species, belonging to 178 genera distributed over 77 families being used in Kashmir and there are many plants which are not being paid due attention [10].* Artemisia *is a widespread and varied genus of the family Asteraceae with great therapeutic and economic importance. It has greater than 500 species [11, 12].* Artemisia amygdalina *is a species of the family Asteraceae having great therapeutic and economic importance*. Artemisia amygdalina* commonly known as “Veer Teethwan” is an erect, up to 1.5 m tall perennial herb. Many stems arise from the base which are shallow to deeply grooved, glabrous, with hairy younger shoots in this plant [13].* Artemisia amygdalina* is a valuable ethnomedicinal angiosperm of Kashmiri ecosystem; it is confined to specific belts of subalpine region in Kashmir [14]. Various antidiabetic plants have been worked out and one such medicinal plant having folklore claims being used in diabetes is* Artemisia amygdalina*.* Artemisia amygdalina* is a widely used medicinal plant in folk medicine [11, 15]. The plant has been reported to have antioxidant potential [16], free radical scavenging activity [13], and anti-inflammatory activity [17]. It also has other pharmacological actions, such as protecting liver,lowering the blood pressure, eliminating fever and sedation, and is used for gastrointestinal ailments [15, 18]. The hexane fraction of* Artemisia amygdalina* has been reported to have potent cytotoxic activity [19]. The active principles in this plant are the terpenes, p-cymene, and 1,8-cineole [20]. Six cytotoxic constituents, namely, ergostadien-3-ol (1), ludartin (2), 5-hydroxy-6,7,3,4-tetramethoxyflavone (3) (from shoot) and trans-matricaria ester (4), diacetylenic spiroenol ether (5), and cis-matricaria ester (6) (from root), have been isolated [19]. In view of its wide ethnomedicinal values, folklore claims, and reported activities, the plant was validated for its antidiabetic activity.

## 2. Materials and Methodology

### 2.1. Plant Material and Extraction

The plant material was collected from the local areas of Kashmir and was identified by the Centre of Taxonomy, University of Kashmir. Sample specimen (voucher specimen number 1803-KASH) was deposited in the herbarium of Centre of Taxonomy, University of Kashmir. The whole plant was used for the extraction. The plant material (whole plant) was completely shade-dried and coarsely ground. The extracts were prepared by continuous hot extraction using petroleum ether, ethyl acetate, methanol and hydroethanol (1 : 1) as solvents. Extracts obtained were concentrated, dried and kept in desiccators for further use. The yields of the petroleum ether, ethyl acetate, methanol, and hydroethanolic extracts were 2.8, 3.2, 5.8, and 6.3% (w/w), respectively.

### 2.2. Experimental Animals

Albino Wistar rats weighing 120–150 g of either sex were selected for the study. They were fed a standard rat pellet and water from Reverse Osmosis Purifier (Kent). Research on animals was conducted in accordance with the guidelines of the Committee for the Purpose of Control and Supervision of Experiments on Animals (CPCSEA) as the institute has CPCSEA registration (Reg. number 927/GO/c/06/CPCSEA). The experimental protocol was approved by the Institutional Animal Ethics Committee of the Regional Research Institute of Unani Medicine, Srinagar, Jammu and Kashmir, India.

### 2.3. Acute Oral Toxicity Study of the Crude Extracts of* Artemisia amygdalina*

Acute oral toxicity study was performed for the extracts of* Artemisia amygdalina* according to the guidelines of the Organisation for Economic Cooperation and Development (OECD) [21]. The rats were kept on fasting overnight, being provided only water prior to oral dosing. Then the extract was administered orally at different dose levels, that is, 100, 200, 500, 1000, 1500, and 2000 mg/kg of body weight. The rats were observed continuously for 24 h for behavioural and any adverse change and thereafter for any lethality.

### 2.4. Experimental Design

All the animals were randomly divided into several groups with five animals in each, serving as normal (nondiabetic), diabetic control, diabetic treated with different extracts, and diabetic reference control, that is, glibenclamide. Glibenclamide was given at a dose of 3 mg/kg of body weight [22, 23]. The oral administration of crude extracts (different extracts with different concentrations) was continued once daily at the same time for 14 days. Body weight and blood glucose levels were estimated on the 0th, 7th, and 14th day of treatment [24].

### 2.5. Induction of Diabetes

Diabetes was induced in albino Wistar rats by a single intraperitoneal (i.p.) injection of 50 mg/kg of streptozotocin (STZ), reconstituted in freshly prepared normal saline (0.9% W/V) after overnight fasting. After 72 h of STZ administration, glucose levels were measured in blood samples collected from retroorbital sinus of rats. Rats with fasting serum glucose levels more than 200 mg/dL were considered diabetic and selected for further study [25, 26].

### 2.6. Assessment of Effects of Extracts on Biochemical Parameters

Oral administration with plant extracts was started 72 h after streptozotocin injection in diabetic rats while normal group and diabetic control group were administered only with vehicle. The rats were sacrificed after 14 days after anesthetising them using isoflurane and blood was collected on the termination day from dorsal vena cava by opening the abdomen. Serum was collected and analyzed for glucose, cholesterol, triglycerides, LDL, SGOT, SGPT, ALP, creatinine, and total protein estimation by using Automatic Biochemistry Analyser (Erba; XL-640).

### 2.7. Effect of Extracts on Body Weight, Feed Consumption, and Water Consumption

The effect of the extracts (hydroethanolic and methanolic) on parameters like body weight, feed consumption, and water consumption were determined and recorded during the study period.

### 2.8. Histopathological Studies

On the 14th day, all the animals were sacrificed and pancreas was removed. Pancreatic samples were taken for histopathology and the tissue samples were processed in tissue processor (Leica made). Pancreatic sections were stained with haematoxylin and eosin Y (H/E) dyes. The sections of pancreas were observed under light microscope (Olympus) for histopathological study.

### 2.9. Oral Glucose Tolerance Test

The oral glucose tolerance test was performed in overnight fasted normal rats [27]. Healthy rats were randomly selected and distributed into six groups (*n* = 5). One group (normal) was administered R.O. water, four groups were given orally extracts of* Artemisia amygdalina* (petroleum ether, ethyl acetate, methanolic, and hydroethanolic each at a dose level of 500 mg/kg of b. w, resp.) and the sixth group was given glibenclamide (3 mg/kg). Glucose (2 g/kg) was fed 1 h after the administration of R.O. water, extracts, and glibenclamide. Blood was collected from the retroorbital sinus under isoflurane inhalation at 0, 30, 60, 90, and 120 minutes (min) of glucose administration and glucose levels were estimated.

### 2.10. Preliminary Phytochemical Tests

The crude methanolic extract, hydroethanolic, ethyl acetate and petroleum ether fractions of* Artemisia amygdalina* were subjected to qualitative tests for identification of different constituents like alkaloids, flavonoids, terpenoids, phenolics, anthraquinones, glycosides, saponins and tannins by using standard qualitative methods described by Trease and Evans [28].

### 2.11. Statistical Analysis

All the values of body weight, fasting serum glucose, and biochemical estimations were expressed as mean ± SEM and ANOVA was carried out followed by post-Dunnett's *t*-test using SPSS 16.0 statistical software. Differences between groups were considered significant at *P* < 0.01 levels.

## 3. Results 

### 3.1. Preliminary Phytochemical Analysis of Different Fractions of* Artemisia amygdalina*

Preliminary screening of methanolic extracts revealed the presence of alkaloids, phenolics, and glycosides. The hydroethanolic extract mainly showed the presence of flavonoids along with phenolics, tannins, and alkaloids while petroleum ether extract showed the presence of terpenes and steroids. The ethyl acetate extract was found to have contents of carbohydrates, glycosides, flavonoids, and terpenoids.

### 3.2. Acute Oral Toxicity Testing

The extracts were found to be safe up to the dose level of 2000 mg/kg of body weight in rats. The extracts did not induce any toxicological effect in any rat. There was no lethality found by oral administration of any extracts of* Artemisia amygdalina*.

### 3.3. Antihyperglycaemic Effect of* Artemisia amygdalina*

The effect of extracts of* Artemisia amygdalina* and glibenclamide on serum glucose levels in normal, diabetic, and extract treated rats is presented in Table 1(a). The highest percent variation in fasting glucose levels was shown by hydroethanolic extract (54.07%), followed by methanolic extract (43.93%). The standard reference drug glibenclamide (3 mg/kg b. w) was found to decrease fasting glucose levels by 48.09% after 14 days of treatment. The other two extracts, that is, pet. ether and ethyl acetate, also showed inhibitory effect on glucose levels but were not considered for further studies. The highly bioactive fractions were tested for dose dependence and were observed to show increased antihyperglycaemic activity with increase in dose. The hydroethanolic fraction showed a more significant effect in decreasing blood glucose levels than the methanolic fraction and the maximum % variation observed at 500 mg/kg b. w was 56.88 while the % variation at the same dose in methanolic fraction was 45.09 (Table 1(b)).

### 3.4. Effect of Extracts of* Artemisia amygdalina *and Glibenclamide on Various Biochemical Parameters in Rats

The extracts of* Artemisia amygdalina *hydroethanolic extract (500 mg/kg b. w) and methanolic extract (500 mg/kg b. w) significantly lowered the levels of cholesterol, triglycerides, LDL, and creatinine in diabetic rats when compared with the diabetic control group (Table 2). Total protein was found to be lowered in diabetic control group, while it was found to be elevated in the extract- and glibenclamide treated diabetic rats. The extracts were also shown to significantly lower the enzymatic activity of liver marker enzymes (SGPT, SGOT, and ALP) in diabetic rats as represented in Table 3.

### 3.5. Effect of Extracts of* Artemisia amygdalina* and Glibenclamide on Feed Consumption and Water Consumption in Rats

The extract treated rats (hydroethanolic and methanolic extract at a dose level of 500 mg/kg b. w) and glibenclamide treated rats (3 mg/kg b. w) significantly overcame the symptoms of diabetes, that is, polyphagia and polydipsia. The extract- and glibenclamide treated rats consumed less water and feed when compared with the diabetic control ones.  Table 4 shows the effect of extracts and glibenclamide on feed and water consumption in rats.

### 3.6. Effect of Extracts of* Artemisia amygdalina* and Glibenclamide on Body Weight in Rats

The body weight of rats belonging to diabetic control group was drastically decreased upon the induction of diabetes. The extract- and glibenclamide treated rats were found to gain body weight significantly when compared with the diabetic control group as shown in Table 5.

### 3.7. Histopathology

**Figure 1:**
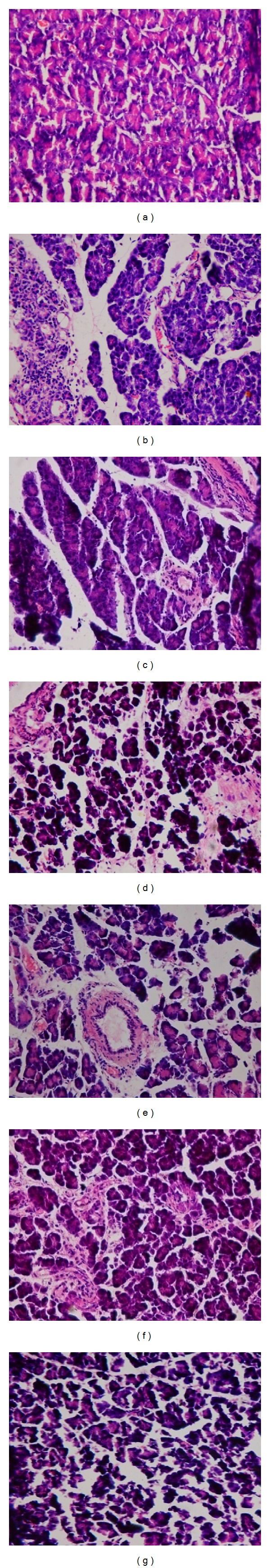
(a) Normal acini and normal cellular population in the islets of Langerhans in pancreas of vehicle-treated rats. (b) Extensive damage to the islets of Langerhans and reduced dimensions of islets. (g) Restoration of normal cellular population size of islets with hyperplasia by glibenclamide. The partial restoration of normal cellular population and enlarged size of **β**-cells with hyperplasia are shown by hydroethanolic extract (250 mg/kg and 500 kg/kg—(c) and (d), resp.) and methanolic extracts (250 mg/kg and 500 mg/kg—(e) and (f), resp.).

Administration of streptozotocin decreased the number of *β*-cells. With the result, the observed mean pancreatic weight of untreated diabetic group was less compared to the mean weight of pancreas of normal (nondiabetic) and diabetic treated groups. The sections from the untreated diabetic group demonstrated shrunken islets of Langerhans with degenerative necrosis. There was an increased vacuolation (Figure 1(b)). In the extract treated rats (Figures 1(c), 1(d), 1(e), and 1(f)) islets of Langerhans appeared less shrunken as compared to those of untreated diabetic group. The histological appearance of the pancreatic islet cells of the control was normal (Figure 1(a)). The morphology of the pancreas of* Artemisia amygdalina *extracts (hydroethanolic and methanolic) treated diabetic rats revealed remarkable improvement in the islets of Langerhans. There was an increase in the islet cellular density, with an increase in granulation, and vacuolation was reduced or absent in many islets (Figures 1(c), 1(d), 1(e), and 1(f)). The histological study of pancreatic sections of glibenclamide treated group resembled extract treated groups in shape, size, and texture (Figure 1(g)).

### 3.8. Oral Glucose Tolerance Test (OGTT)

Table 6(a) shows the OGTT study; blood glucose concentration in all groups reached peak levels after 30 min of glucose administration (2 g/kg) and then began to decrease. As compared to normal group, the glucose levels of experimental rats treated with extracts and glibenclamide showed a very steep reduction. Hydroethanolic extract (500 mg/kg of b. w) showed more significant antihyperglycemic activity than other extracts and glibenclamide treated rats. Hydroethanolic and methanolic extracts were found to lower the glucose levels in a dose-dependent pattern (Table 6(b)).

## 4. Discussion

Many Indian medicinal plants are reported to be useful in diabetes mellitus and many species of genus* Artemisia* have been reported to have antidiabetic activity [29]. In* Artemisia indica* the hydromethanolic crude extract at a dose of 200 and 400 mg/kg b. w and chloroform fraction (200 mg/kg b. w) administered orally for 15 days showed a significant reduction in blood glucose level [30]. Similar results were observed in our study that was carried out on* Artemisia amygdalina *where hydroethanolic and methanolic extract produced a significantdecrease in the serum glucose level at a dose of 500 mg/kg. The extracts also showed increased dose-dependent antihyperglycaemic effect. The results also correlate with the study carried on* Artemisia judaica*, where the bioactive principles found were the flavonoids [31]. In other similar studies carried out on* Artemisia sieberi, Artemisia pallens, *and* Artemisia herba-alba,* treatment of diabetic rats with the fractions obtained from these plants significantly reduced the blood glucose levels [30, 32–34]. These studies suggest the commonness of bioactive principles among these related species, thus making them effective in the treatment of diabetes.

Since liver is the central metabolic organ in body responsible for glucose and lipid homeostasis, diabetes leads to hepatic dysfunction. Streptozotocin induced diabetes in rats causes the elevated activities of liver marker enzymes (SGPT, SGOT, and ALP) due to the reported destruction of hepatocytes [35]. Rats orally treated with the extracts of* Artemisia amygdalina *(hydroethanolic and methanolic extracts each at a dose level of 500 mg/kg of b. w) were found to have significantly reduced activities of these enzymes, thus indicating less damage to hepatocytes.

Streptozotocin induced diabetic rats are associated with hyperlipidemia and increased levels of serum creatinine [36]. However the extract treated rats (hydroethanolic and methanolic extracts each at a dose level of 500 mg/kg of b. w) showed reduced levels of cholesterol, triglycerides, LDL, and serum creatinine when compared with the diabetic control group. The lowering of these lipid substances and serum creatinine in the blood of treated rats is presumed mainly to be a manifestation of lowering of blood glucose level.

The serum protein levels in diabetic rats were reduced as compared to normal group, while the serum protein levels of treated rats (hydroethanolic and methanolic extracts each at a dose level of 500 mg/kg of b. w) were found to be higher than those of diabetic group indicating lowered protein degradation. There was a significant reduction in the feed and water consumption in diabetic rats treated with hydroethanolic and methanolic extracts of* Artemisia amygdalina *(each at a dose level of 500 mg/kg of b. w) when compared with the diabetic control group. The decrease in feed and water consumption may be attributed to the decrease in protein degradation and improved glycemic control as is obvious from the results stated earlier. The body weight among the rats administered with the extracts of* Artemisia amygdalina *was found to be in an increasing fashion possibly due to the reduction in lowering of glucose levels thus sparing the body fat and muscle protein which otherwise are utilised in diabetic rats.

Streptozotocin (STZ) administration generally causes the destruction of *β*-cells after three days and reaches its peak at three to four weeks in rats [37]. *β*-cells are particularly sensitive to damage by nitric oxide and free radicals because of their low levels of free radical scavenging enzymes [38]. The results of this present study indicate that the extracts of* Artemisia amygdalina* may lead to the regeneration/proliferation of the pancreatic *β*-cells possibly due to the prevention of free radical formation induced by STZ. Since pancreas contains stable (quiescent) *β*-cells which have the regenerative capacity, the surviving cells proliferate by replication to supplicate the lost cells [39]. New pancreatic *β*-cells can be formed by neogenesis or by replication of the preexisting differentiated cells [40]; hence it is assumed from the study that the extracts of* Artemisia amygdalina* are also responsible for the proliferation of *β*-cells, as there are already reports showing extracts of other medicinal plants which have a *β*-cell regenerative potential [41, 42].

The antidiabetic potential of the different extracts of* Artemisia amygdalina *may be due the presence of any of the secondary metabolites (flavonoids, alkaloids, phenolics, glycosides, and terpenes) present in variedconcentrations in* Artemisia amygdalina.* These secondary metabolites are reported to possess antidiabetic potential differently and are the suggested reason for the difference in activities of these extracts [43–48]. Many of the flavonoids have been found to possess the antidiabetic potential [49]. We also suggest the potent antidiabetic potential of hydroethanolic extract because of the presence of flavonoid compound(s) in it.

The improved glycemic control in oral glucose tolerance tests by the extracts of* Artemisia amygdalina *shows that the extracts also lower the blood glucose levels even in normal rats. The effect of lowering blood glucose levels in normal rats may be due to the increased efficiency of the peripheral tissues for the uptake of glucose from blood. Thus the extracts can also be useful in patients with type II diabetes and need further detailed studies for the validation of these results.

## 5. Conclusion

The common symptoms of diabetes, that is, polyphagia, polydipsia, and weight loss, were found to be lessened by the extracts of* Artemisia amygdalina* (hydroethanolic and methanolic extracts each at a dose level of 500 mg/kg of b. w) in diabetic rats. The extracts significantly reduced fasting glucose levels in diabetic rats and also reduced the lipid profile parameters in diabetic rats. The extracts were found significantly decreasing the activities of SGPT, SGOT, and ALP in diabetic rats. In conclusion, our histopathological investigation along with the biochemical evaluations suggests the strong antidiabetic potential of* Artemisia amygdalina.* The results observed show the effect on both the pancreatic *β*-cells and the blood glucose level. Further mechanistic studies are required to suggest the appropriate mechanism for the antidiabetic effect of the plant.

## Figures and Tables

**Table:** (a)

Groups	Serum glucose (mg/dL)			
	0th day	7th day	14th day	% variation
Normal	82.32 ± 0.99	85.43 ± 1.39	83.57 ± 2.22	−1.51
Diabetic control	360.14 ± 5.06	387.58 ± 3.28	410.13 ± 2.48	−13.88
Pet Ether extract (500 mg/kg b. w)	350.55 ± 3.26	309.87 ± 2.85	266.57 ± 4.10	23.95
Hydroethanolic extract (500 mg/kg b. w)	386.47 ± 5.51	232.51 ± 3.82**	177.49 ± 2.00**	54.07
Methanolic extract (500 mg/kg b. w)	359.54 ± 3.33	284.65 ± 2.83**	201.59 ± 3.38**	43.93
Ethyl Acetate extract (500 mg/kg b. w)	371.35 ± 2.92	325.43 ± 6.43	260.53 ± 2.94	29.84
Glibenclamide (3 mg/kg b. w)	365.9 ± 3.97	248.78 ± 4.16**	189.92 ± 3.07**	48.09

The values are expressed as mean ± SEM; *n* = 5 in each group. **P < 0.01 as compared with normal at the same time (one-way ANOVA followed by Dunnett's multiple comparison test).

**Table:** (b)

Groups	Serum Glucose (mg/dL)			
	0th day	7th day	14th day	% variation
Normal	85.66 ± 1.09	84.86 ± 1.13	84.12 ± 2.25	1.80
Diabetic control	375.41 ± 4.62	390.82 ± 4.75	419.52 ± 3.94	−11.75
Hydroethanolic extract (50 mg/kg b. w)	380.76 ± 5.24	366.27 ± 4.55	352.78 ± 5.84	7.35
Hydroethanolic extract (100 mg/kg b. w)	355.15 ± 4.48	330.85 ± 6.3	288.54 ± 3.96	18.76
Hydroethanolic extract (250 mg/kg b. w)	370.59 ± 5.23	290.36 ± 6.34**	238.45 ± 7.46**	35.66
Hydroethanolic extract (500 mg/kg b. w)	377.81 ± 5.45	227.55 ± 4.36**	162.92 ± 3.85**	56.88
Methanolic extract (50 mg/kg b. w)	358.96 ± 6.94	350.12 ± 7.24	344.4 ± 5.74	4.06
Methanolic extract (100 mg/kg b. w)	366.85 ± 5.89	340.53 ± 7.84	328.12 ± 4.85	10.56
Methanolic extract (250 mg/kg b. w)	390.61 ± 6.37	358.24 ± 5.67	295.48 ± 6.13	24.35
Methanolic extract (500 mg/kg b. w)	365.37 ± 8.7	278.42 ± 4.75**	200.64 ± 5.82**	45.09
Glibenclamide (3 mg/kg b. w)	372.27 ± 4.86	258.87 ± 5.64**	195.25 ± 4.37**	47.55

The values are expressed as mean ± SEM; *n* = 5 in each group. **P < 0.01 as compared with normal at the same time (one-way ANOVA followed by Dunnett's multiple comparison test).

**Table 2:** Effect of extracts of *Artemisia amygdalina* and glibenclamide on various biochemical parameters in rats.

Groups	Cholesterol (mg/dL)	Triglycerides (mg/dL)	LDL (mg/dL)	Total protein (g/dL)	Creatinine (mg/dL)
Normal	50.59 ± 2.77	62.83 ± 2.46	83.35 ± 3.22	7.11 ± 0.30	0.69 ± 0.05
Diabetic control	149.33 ± 6.93	120.83 ± 4.96	189.38 ± 5.61	4.20 ± 0.44	1.98 ± 0.09
Hydroethanolic extract (250 mg/kg b. w)	84.32 ± 3.67	95.74 ± 5.54	132.45 ± 3.35	5.75 ± 0.29	1.18 ± 0.05
Hydroethanolic extract (500 mg/kg b. w)	64.83 ± 2.19**	75.66 ± 4.87**	101.36 ± 2.95**	6.12 ± 0.32	0.88 ± 0.08**
Methanolic extract (250 mg/kg b. w)	88.12 ± 3.79	98.27 ± 5.28	142.77 ± 4.23	5.15 ± 0.26	1.07 ± 0.06
Methanolic extract (500 mg/kg b. w)	73.35 ± 2.92**	84.43 ± 6.44**	128.54 ± 3.46**	6.67 ± 0.28**	0.97 ± 0.07**
Glibenclamide (3 mg/kg b. w)	60.83 ± 2.73**	78.00 ± 2.73**	92.37 ± 3.67**	7.03 ± 0.21**	0.78 ± 0.04**

The values are expressed as mean ± SEM; *n* = 5 in each group. **P < 0.01 as compared with diabetic control at the same time (one-way ANOVA followed by Dunnett's multiple comparison test).

**Table 3:** Effect of extracts of *Artemisia amygdalina *and glibenclamideon liver marker enzymes of streptozotocin induced diabetic rats.

Groups	SGOT (U/L)	SGPT (U/L)	ALP (U/L)
Normal	115.00 ± 5.14	95.23 ± 6.75	175.67 ± 8.68
Diabetic control	243.00 ± 9.57	209.16 ± 6.35	296.17 ± 7.85
Hydroethanolic extract (250 mg/kg b. w)	164.31 ± 6.53	148.76 ± 7.69	232.17 ± 6.82
Hydroethanolic extract (500 mg/kg b. w)	129.66 ± 8.08**	112.83 ± 7.11**	201.66 ± 7.94**
Methanolic extract (250 mg/kg b. w)	174.76 ± 4.83	177.62 ± 7.71	229.77 ± 6.18
Methanolic extract (500 mg/kg b. w)	138.53 ± 2.94**	121.85 ± 6.69**	219.53 ± 7.37
Glibenclamide (3 mg/kg b. w)	119.50 ± 4.43**	103.73 ± 3.00**	198.50 ± 7.14**

The values are expressed as mean ± SEM; n = 5 in each group. **P < 0.01 as compared with diabetic control at the same time (one-way ANOVA followed by Dunnett's multiple comparison test).

**Table 4:** Effect of the extracts of *Artemisia amygdalina* and glibenclamideon feed intake and fluid intake of the rats.

Groups	Water consumption (mL/day)	Food consumption (g/day)
Normal (water)	24 ± 4.02	19.34 ± 2.36
Diabetic (water)	65 ± 7.60	29.67 ± 1.23
Hydroethanolic extract (250 mg/kg b. w)	46 ± 5.37	25.33 ± 3.30
Hydroethanolic extract (500 mg/kg b. w)	39 ± 7.16**	23.56 ± 3.42**
Methanol extract (250 mg/kg b. w)	49 ± 4.02	24.24 ± 2.60
Methanol extract (500 mg/kg b. w)	44 ± 4.92**	22.87 ± 2.01**
Glibenclamide (3 mg/kg b. w)	44 ± 6.71**	22.33 ± 1.23**

The values are expressed as mean ± SEM; *n* = 5 in each group. **P < 0.01 as compared with diabetic control at the same time (one-way ANOVA followed by Dunnett's multiple comparison test).

**Table 5:** Effect of extracts of *Artemisia amygdalina* and glibenclamideon body weight in streptozotocin induced diabetic rats.

Groups	Body weight (g)			
	0th day	7th day	14th day	% variation
Normal	120 ± 1.79	135 ± 3.13	155 ± 2.68	29.16
Diabetic control	125 ± 1.34	118 ± 1.79	107 ± 3.13	−14.4
Hydroethanolic extract (250 mg/kg b. w)	127 ± 2.24	132 ± 2.24	139 ± 3.58**	9.44
Hydroethanolic extract (500 mg/kg b. w)	122 ± 2.68	128 ± 3.13	135 ± 2.24**	10.65
Methanolic extract (250 mg/kg b. w)	125 ± 3.13	130 ± 3.58	137 ± 4.92**	9.6
Methanolic extract (500 mg/kg b. w)	123 ± 2.24	130 ± 3.58	140 ± 5.37**	13.82
Glibenclamide (3 mg/kg b. w)	125 ± 1.79	133 ± 2.68	142 ± 3.13**	13.6

The values are expressed as mean ± SEM; *n* = 5 in each group. **P < 0.01 as compared with diabetic control at the same time (one-way ANOVA followed by Dunnett's multiple comparison test).

**Table:** (a)

Groups	Blood glucose level (mg/dL)					
	0 min	30 min	60 min	90 min	120 min	% variation
Normal	82.35 ± 3.44	147.58 ± 4.11	133.60 ± 5.05	125.34 ± 6.40	118.36 ± 3.53	43.72
Pet. ether extract (500 mg/kg b. w)	83.14 ± 2.82	138.58 ± 6.84	127.13 ± 2.46	118.93 ± 5.72	90.57 ± 3.44	8.93
Ethyl acetate extract (500 mg/kg b. w)	80.76 ± 2.10	142.48 ± 3.53	125.71 ± 6.40	112.91 ± 5.90	94.64 ± 6.35	17.18
Hydroethanolic extract (500 mg/kg b. w)	83.245 ± 3.71	110.68 ± 3.00**	97.79 ± 3.49**	89.51 ± 4.20**	79.51 ± 4.52**	-4.48
Methanolic extract (500 mg/kg b. w)	80.47 ± 3.22	122.27 ± 4.20	112.72 ± 4.38	98.61 ± 5.99	86.44 ± 4.65**	7.414
Glibenclamide (3 mg/kg b. w)	81.89 ± 3.00	118.82 ± 3.85**	107.82 ± 3.67**	98.24 ± 4.29**	82.56 ± 4.11**	0.81

The values are expressed as mean ± SEM; *n* = 5 in each group. **P < 0.01 as compared with normal at the same time (one-way ANOVA followed by Dunnett's multiple comparison test).

**Table:** (b)

Groups	Blood glucose level (mg/dL)					
	0 min	30 min	60 min	90 min	120 min	% variation
Normal	81.25 ± 2.97	149.49 ± 3.24	136.64 ± 4.22	128.44 ± 5.53	115.42 ± 6.30	42.05
Hydroethanolic extract (50 mg/kg b. w)	82.41 ± 3.23	145.45 ± 4.67	131.24 ± 2.35	122.46 ± 4.86	112.25 ± 4.33	36.20
Hydroethanolic extract (100 mg/kg b. w)	83.67 ± 4.15	132.56 ± 4.32	120.18 ± 4.50	110.16 ± 4.34	101.14 ± 5.36	20.88
Hydroethanolic extract (250 mg/kg b. w)	81.54 ± 5.25	121.24 ± 2.96**	107.73 ± 4.39**	96.64 ± 3.74**	87.15 ± 2.54**	6.88
Hydroethanolic extract (500 mg/kg b. w)	82.42 ± 4.64	108.68 ± 3.38**	96.21 ± 3.44**	83.31 ± 4.72**	72.15 ± 5.42**	221212.46
Methanolic extract (50 mg/kg b. w)	83.66 ± 4.12	150.52 ± 3.35	140.88 ± 3.48	128.25 ± 2.78	118.26 ± 3.35	41.35
Methanolic extract (100 mg/kg b. w)	82.52 ± 4.45	141.65 ± 3.22	129.42 ± 3.68	116.27 ± 3.85	105.51 ± 5.22	27.86
Methanolic extract (250 mg/kg b. w)	83.24 ± 5.24	131.57 ± 4.38	116.21 ± 5.12	106.35 ± 4.62	95.23 ± 4.95**	14.40
Methanolic extract (500 mg/kg b. w)	82.57 ± 6.35	120.09 ± 5.14	103.41 ± 2.56	92.35 ± 3.44	81.13 ± 2.55**	1.74
Glibenclamide (3 mg/kg b. w)	80.68 ± 2.75	116.78 ± 5.83**	104.23 ± 6.73**	94.16 ± 5.25**	83.25 ± 4.75**	3.18

The values are expressed as mean ± SEM; *n* = 5 in each group. **P < 0.01 as compared with normal at the same time (one-way ANOVA followed by Dunnett's multiple comparison test).
